# On the Psychology of TikTok Use: A First Glimpse From Empirical Findings

**DOI:** 10.3389/fpubh.2021.641673

**Published:** 2021-03-16

**Authors:** Christian Montag, Haibo Yang, Jon D. Elhai

**Affiliations:** ^1^Department of Molecular Psychology, Institute of Psychology and Education, Ulm University, Ulm, Germany; ^2^The Clinical Hospital of Chengdu Brain Science Institute, MOE Key Lab for Neuroinformation, University of Electronic Science and Technology of China, Chengdu, China; ^3^Faculty of Psychology, Tianjin Normal University, Academy of Psychology and Behavior, Tianjin, China; ^4^Department of Psychology, University of Toledo, Toledo, OH, United States; ^5^Department of Psychiatry, University of Toledo, Toledo, OH, United States

**Keywords:** TikTok, DouYin, musical.ly, personality, uses and gratification, social media, social media addiction, problematic social media use

## Abstract

TikTok (in Chinese: DouYin; formerly known as musical.ly) currently represents one of the most successful Chinese social media applications in the world. Since its founding in September 2016, TikTok has seen widespread distribution, in particular, attracting young users to engage in viewing, creating, and commenting on “LipSync-Videos” on the app. Despite its success in terms of user numbers, psychological studies aiming at an understanding of TikTok use are scarce. This narrative review provides a comprehensive overview on the small empirical literature available thus far. In particular, insights from uses and gratification theory in the realm of TikTok are highlighted, and we also discuss aspects of the TikTok platform design. Given the many unexplored research questions related to TikTok use, it is high time to strengthen research efforts to better understand TikTok use and whether certain aspects of its use result in detrimental behavioral effects. In light of user characteristics of the TikTok platform, this research is highly relevant because TikTok users are often adolescents and therefore from a group of potentially vulnerable individuals.

## Background

Musical.ly was founded in September 2016 by Zhang Yiming. Beijing Bytedance Technology acquired the application musical.ly in November 2017 and renamed the app to TikTok. In a short time period, this application became the most successful app from Chinese origin in terms of global distribution (1). As of November 2020, 800 million monthly users have been reported^1^, and 738 million first-time installs in 2019 have been estimated^2^. TikTok use is allowed for those 13 years or older, but direct messaging between users is allowed only for those 16 or older (in order to protect young users from grooming)^3^. In China, the main users of TikTok are under 35 years old (81.68% (2)). Meanwhile, to protect children and adolescents from unsuitable content (such as smoking, drinking, or rude language), TikTok's engineers also developed a version of the app, which filters inappropriate content for young users (2). Of note, at the moment of writing, the app operates as TikTok on the global market and as DouYin on the Chinese market (3). Similarities and differences of the twin apps are further described with a content analysis by Sun et al. (4).

The TikTok application available for Android and Apple smartphones enables creation of short videos where users can perform playback-videos to diverse pop-songs, to name one very prominent feature of the platform. These so-called “LipSync-Videos” can be shared with other users, downloaded for non-commercial purposes, commented upon and of course attached with a “Like.” Not only are playback-videos uploaded on TikTok but also users view a large amount of video content. Users can also call out for “challenges,” where they define which performance should be created by many users. As a consequence, TikTok users imitate the content or interact with the original video.

As the large user numbers in a very short time-window demonstrate, TikTok not only represents a global phenomenon but also has been criticized with respect to data protection issues/privacy (5, 6), spreading hate (7) and might serve as a platform engendering cyberbullying (8, 9). Given the many young users of this platform (e.g., 81.68% of China users of Tiktok are under 35 years old—see above, and 32.5% of the US users are 19 years old and younger)^4^, it is of particular relevance to better understand the motivation to use TikTok, alongside related topics. Such an understanding might also be relevant because recent research suggests that TikTok can be a potent channel to inform young persons on health-relevant information (10–12), on official information release from the government (13), political discussions (14), tourism content (15), live online sales (16), and even educational content (17). There even have been video-posts analyzed in a scientific paper related to radiology (18). Clearly, young TikTok users are also confronted with harmful health content, including smoking of e-cigarettes (19). Moreover, the health information learned from TikTok videos often does not meet necessary standards—as is discussed in a paper on acne (20). Finally, there arises the problem that while creating content, children's/adolescent's private home bedrooms from which they create TikTok videos become visible to the world, posing privacy intrusions (21). The many obviously negative aspects of TikTok use are in itself important further research leads. From a psychological perspective, we take a different path with the present review and try to better understand why people use TikTok, who uses the platform, and also how people use TikTok.

## Why do People Use TikTok?

This question can be answered from different perspectives. One perspective providing an initial answer and—likely being true for most social media services—has been put forward by Montag and Hegelich (22). Social media companies have created services being highly immersive, aiming to capture the attention of users as long as possible (23). As a result of a prolonged user stay, social media companies obtain deep insights into psychological features of their users (24), which can be used for microtargeting purposes (25). Such immersive platform design also likely drives users with certain characteristics into problematic social media use (26) or problematic TikTok use (addictive-like behavior), but this aspect relating to TikTok use is understudied. Nevertheless, reinforcement of TikTok usage is also very likely reached by design-elements such as “Likes” (27), personalized and endless content available (23). TikTok's “For You”-Page (the landing page) learns quickly via artificial intelligence what users like, which likely results in longer TikTok use than a user intended, which may cause smartphone TikTok-related addictive behavior (2). This said, these ideas put forward still need to be confirmed by empirical studies dealing exclusively with TikTok. In this realm, an interesting research piece recently investigated less studied variables such as first-person camera views, but also humor on key variables such as immersion and entertainment on the TikTok platform (28), again all of relevance to prolong user stay.

The other perspective one could choose to address why people use TikTok stems from uses and gratification theory (29, 30). The simple idea of this highly influential theory is that use of certain media can result in gratification of a person's needs (30), and only if relevant needs of a person are gratified by particular media, users will continue media use—here digital platform or social media use.

A recent paper by Bucknell Bossen and Kottasz (31) provided insight that, in particular, gratification of entertainment/affective needs was the most relevant driver to understand a range of behaviors on TikTok, including passive consumption of content, but also creating content and interacting with others. In particular, the authors summarized that TikTok participation was motivated by needs to expand one's social network, seek fame, and express oneself creatively. Recent work by Omar and Dequan (32) also applied uses and gratification theory to better understand TikTok use. In their work, especially the need for escapism predicted TikTok content consumption, whereas self-expression was linked to both participating and producing behavior. A study by Shao and Lee (33) not only applied uses and gratification theory to understand TikTok use but also shed light on TikTok use satisfaction and the intention to further use TikTok. In line with findings from the already mentioned works, entertainment/information alongside communication and self-expression were discussed as relevant use motives (needs to be satisfied by TikTok use). Satisfaction with TikTok was investigated as a mediator between different motives to use TikTok and to continue TikTok use. We also mention recent work being unable to link TikTok use to well-being, whether in a positive or negative way (34). Finally, Wang et al. (35) underlined the overall relevance of uses and gratification theory to understand TikTok use and presented need variables in cognitive and affective domains as relevant to study, but also personal/social integration and relief of pressure. In this context, we also mention the view of Shao (2) who put forward that, in particular, young people use TikTok for positioning oneself in their peer group and to understand where he/she stands in the peer group. Thus, TikTok is also relevant for identity formation of young persons and obtaining feedback to oneself.

Further theories need to be mentioned, which can explain *why* people are using the TikTok platform: Social Impact Theory and Self-Determination Theory. To our knowledge, these theories have not been sufficiently addressed empirically so far with respect to TikTok use, but are well known to be of relevance to understand social media use in general and are therefore mentioned.

Clearly, an important driver of social media use can be power, hence, reaching out to many and influencing other persons (36). Here, the classic Social Impact Theory (SIT) by Latané (37) tries to understand how to best measure the impact of people on a single individual/individuals. This theory—originating in the pre-social-media-age—gained a lot of visibility with the rise of social media services because, in particular, in the age of filter bubbles, fake news, and misinformation campaigns (38, 39), it is interesting to understand how individual users on social media are socially influenced by others, for instance, in the area of their (political) attitudes. The SIT postulates three highly relevant factors called strength, immediacy, and number (of sources) to predict such a social impact. Ultimately, applying this theory to better understand TikTok use also needs to take into account that users differ in terms of their active and passive use.

The Self-Determination Theory (SDT) has been proposed by Ryan and Deci (40) and belongs to the most influential motivation theories of human behavior. Hence, it clearly can also be used to explain why people are motivated to use a social media service (41, 42). According to SDT, motivated behavior (here using TikTok) should be high, when such a platform enables users to feel competence, autonomy, and being connected with others. Design of the platform can help to trigger related psychological states (e.g., push notifications can trigger fear of missing out, hence, not being connected to significant others) (43); but clearly also, individual differences play a relevant role, and this should be discussed as the next important area in this work. As with the SIT, applying SDT to better understand TikTok use will also need to take into account different kinds of TikTok use. A sense of self-determination might rise to different levels, when users are actively or passively using TikTok—and this also represents an interesting research question.

## Who Uses TikTok and Who Does Not?

The aforementioned statistics show that TikTok users are often young. Bucknell Bossen and Kottasz (31) illustrated that, in particular, young users are also those who seem to be particularly active on the platform, and thus share much information. Given that, in particular, young users often do not foresee consequences of self-disclosure, it is of high importance to better protect this vulnerable group from detrimental aspects of social media use. Beyond age, statistics suggest that more females than males use the platform^5^, something also observed with other platforms (44–46). First, insights from personality psychology provided further information on associations between characteristics of TikTok users and how they use it (see also the next How Do People Use TikTok? section): The widely applied Big Five Personality traits called openness to experience, conscientiousness, extraversion, agreeableness, and neuroticism (acronym OCEAN) were all robustly linked to producing, participating, and consuming behavior on TikTok, with the exception of agreeableness only being linked to consuming behavior (32). Using a hierarchical regression model inserting both personality variables and motives from uses and gratification theory, it became apparent that the latter variables seemed to outweigh the personality variables in their importance to predict TikTok usage. Lu et al. (47) used data from China to investigate individual differences in DouYin (again the Chinese version of TikTok) use. Among others, they observed that people refraining from using DouYin did so out of fear of getting “addicted” to the application [see also (48)]. This needs to be further systematically explored with the Big Five model of personality (or HEXACO, as the personality models dominating modern personality psychology at the moment). Without doubt, it will be also highly important to better understand how the variables of socio-demographics and personality interact on TikTok use, also in the realm of active/passive use of the platform. Active use would describe a high engagement toward the platform including commenting and uploading videos. Passive usage would reflect in browsing and simply consuming videos. The need to distinguish between active and passive use of social media has been also recently empirically supported by Peterka-Bonetta et al. (49).

## How do People Use TikTok?

In the Why Do People Use TikTok? section, we already mentioned that users can passively view content, but also create content or interact with others. Studies comprehensively showing how many and which types of people use TikTok with respect to these behavioral categories are lacking (but TikTok likely has at least some of these insights). A recent review by Kross et al. (50) on “social media (use) and well-being” summarized that several psychological processes such as upward social comparison (perhaps also happening in so-called “challenges” on TikTok) or fear of missing out (43) are related to negative affect and might have detrimental effects on the usage experience and/or TikTok users' lives in general. Overall, the psychological impact of the TikTok platform might also be very likely, in particular, when adolescents often imitate their idols in “LipSync-Videos” (51). The kind of influence of such behavior on the development of one's own identity and self-esteem (self-confidence) (52) will be a matter of important psychological discussion, but it is too early to speculate further on potential psychological effects here, both in the positive or negative direction (53). Moreover, whether such effects will be of positive or negative nature, we mention the importance to not overpathologize everyday life behavior (54).

In sum, much of what we know with respect to platforms such as Instagram, Facebook, WhatsApp, or even WeChat (56) needs to still be investigated in the context of TikTok, to understand if psychological observations made for other social media channels can be transferred “one-on-one” to TikTok. For instance, illustrating differences between social media platforms, Bhandari and Bimo (57) suggested in their analysis of TikTok that in contrast to other platforms, “the crux of interaction is not between users and their social network, but between a user and what we call an ‘algorithmized’ version of self.” Opening TikTok immediately results in being captured by a personalized stream of videos. Therefore, we believe it to be unlikely that all insights from social media research can be easily transferred to TikTok because it is well-known that each social media platform has a unique design also attracting different user groups (45), and they elicit different immersive or “addictive” potential (58). Please note that we use the term “addictive” only in quotation marks, given the ongoing debate on the actual nature of excessive social media use (59, 60). This said, we explicitly mention that the study of problematic social media use represents a very important topic (61), although at the moment, this condition—of relevance for the mental health sciences—is not officially recognized by the World Health Organization. Despite the ongoing controversy, nevertheless, it has been recently pointed out that social media companies are responsible for the well-being of users, too (55).

## Conclusions and Outlook

**Figure 1:**
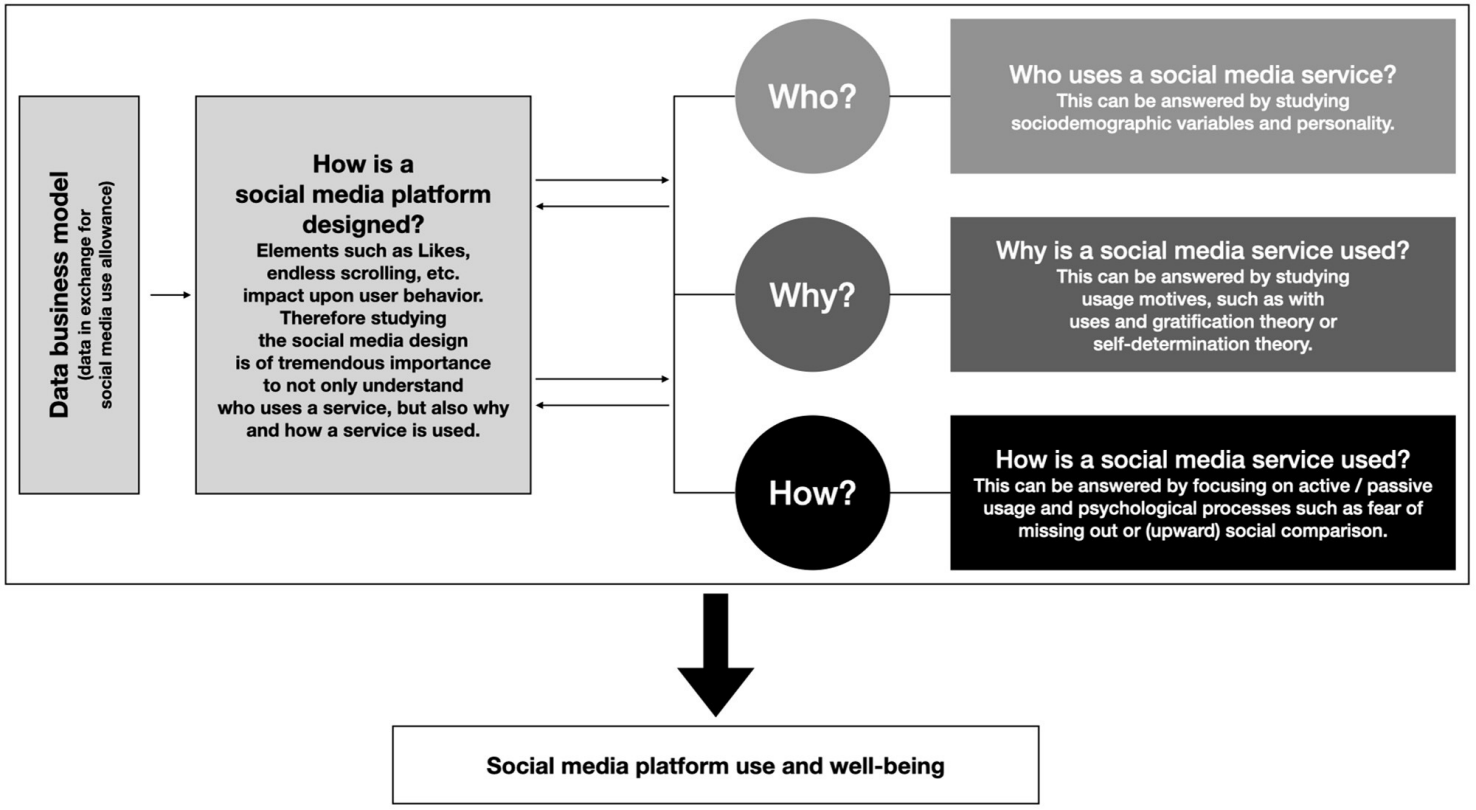
In order to understand the relationship between a social media service such as TikTok and human psychological processes and behavior, one needs to answer the who-, why-, and how-questions, also against the background of the social media platform design. Please note that the platform design itself is driven by the data business model. Social media usage and its association with psychological/behavioral variables such as well-being, online-time, and so on can be best understood by investigating these variables in one model, at best also investigating potential interactions of variables. These ideas have also been described in parts in Montag and Hegelich (22), Kross et al. (50), and Montag et al. (55). The figure does not exclusively mention TikTok because we are convinced that the presented details are true for all research agendas aiming at a better understanding of the relationship between social media use and well-being.

Although user numbers are high and TikTok represents a highly successful social media platform around the globe, we know surprisingly less about psychological mechanisms related to TikTok use. Most research has been carried out so far yielding insights into user motives applying uses and gratification theory. Although this theory is of high importance to understand TikTok use, it is still rather broad and general. In particular, when studying a platform such as TikTok—receiving attention at the moment from a lot of young users—more specific needs or facets of the broad dimensions of uses and gratification theory (such as social usage) being more strongly related to the needs of adolescents might need more focus. One such focus could be a stronger emphasis on the study of self-esteem (62) in the context of TikTok use. Work beyond this area, e.g., investigating potential detrimental aspects, are scarce, but will be important. In particular, we deem this to be true, as TikTok attracts very young users, being more vulnerable to detrimental aspects of social media use (63). We believe that it is also high time for researchers to put research energy in the study of TikTok and to do so in a comprehensive manner. Among others, it needs also to be studied how active and passive use impact on the well-being of the users. This means that the here-discussed *how-, why-*, and *who-*questions need to be studied together in one framework, and this needs to be done against the data business model and its immersive platform design. The key ideas of this review to understand TikTok use and related aspects such as well-being of the users are presented in Figure 1.

## Author Contributions

CM wrote the first draft of this review article. HY screened the Chinese literature and added relevant work from a Chinese perspective to the review. Finally, JDE critically worked over the complete draft. All authors agreed upon the final version of the article.

## Conflict of Interest

The authors declare that the research was conducted in the absence of any commercial or financial relationships that could be construed as a potential conflict of interest.

## Footnotes

^1^https://www.omnicoreagency.com/tiktok-statistics/ (accessed March 9, 2021).

^2^https://www.statista.com/statistics/1089420/tiktok-annual-first-time-installs/ (accessed March 9, 2021).

^3^https://www.heise.de/newsticker/meldung/Ab-16-TikTok-fuehrt-Mindestalter-fuer-Direktnachrichten-ein-4703887.html (accessed March 9, 2021).

^4^https://www.statista.com/statistics/1095186/tiktok-us-users-age/ (accessed March 9, 2021).

^5^https://www.statista.com/statistics/1095196/tiktok-us-age-gender-reach/ (accessed March 9, 2021).
